# Subcutaneous mastectomy in female-to-male transsexuals is associated with higher risk of postoperative bleeding complications

**DOI:** 10.1016/j.jpra.2023.11.004

**Published:** 2023-11-20

**Authors:** Martynas Tamulevicius, Doha Obed, Nadjib Dastagir, Tobias R. Mett, Peter M. Vogt, Khaled Dastagir

**Affiliations:** a Department of Plastic, Aesthetic, Hand and Reconstructive Surgery, Hannover Medical School, Carl-Neuberg-Str. 1, D-30625 Hannover; b Department of Plastic, Aesthetic and Reconstructive Surgery, Evangelical Hospital Goettingen – Weende, An d. Lutter 24, D-37075, Goettingen

**Keywords:** Mastectomy, Female-to-male, Transsexuals, Transgender, Gender reassignment

## Abstract

**Introduction:**

Chest contouring or subcutaneous mastectomy (SCM) in female-to-male (FtM) transgender individuals is the primary surgery in the gender reassignment process. Many authors report high rates of postoperative bleeding in these patients and discuss a possible influence of preoperative hormone therapy. However, there is a lack of data on the analysis between different surgical techniques and postoperative bleeding risk.

**Materials and Methods:**

In this retrospective study, we included 22 FtM transgender individuals who underwent bilateral SCM using 4 different techniques (44 breasts) between June 2014 and September 2023. Postoperative complications regarding surgical techniques and patient demographics were collected and analyzed.

**Results:**

SCM with free nipple grafting was the most commonly used technique (n = 12, 54.5%). The mean operative time was 163.4 ± 49.2 minutes. There were no significant differences in operative time between the surgical techniques (p ≥ 0.20 in all cases). The rate of acute postoperative bleeding was 20.5% (n = 9). Acute postoperative bleeding occurred most frequently in patients who received a semi-circular incision for SCM. There was no significant difference in the rate of acute postoperative bleeding between the different surgical techniques. BMI, breast weight, and duration of surgery were not associated with the rate of acute complications (p > 0.17 in all cases).

**Conclusions:**

Less invasive SCM techniques in FtM transgender individuals are associated with higher postoperative bleeding risk.

## Introduction

Individuals with a gender identity that does not match their physical sex assigned at birth are often described as “gender incongruent,” transgender, or simply “trans” people. The prevalence of the transgender population globally is estimated to be around 0.3-0.5% ^1^. The desire to live and be socially accepted as an opposite gender leads to immense psychological distress and can result in the impairment of social and occupational activities of daily life and, in some cases, even suicide attempts ^2^. Thus, it is crucial to understand disproportionate challenges, specific medical issues, and barriers related to the transgender population. Gender confirmation surgery plays a key role in the treatment of gender dysphoria in these patients^3^.

Transgender individuals suffer from severe psychological distress and therefore require access to gender affirmation services and generally evidence-based healthcare ^2^. In addition to psychological support, a detailed screening for mental and sexually transmittable diseases should be conducted in primary care setting before any gender affirmation surgeries to reduce additional risks ^4^^,^^5^. Moreover, a minimum of 1 year of cross-sex hormonal treatment is recommended before any surgical intervention ^6^.

Female-to-male (FtM) transsexualism is a medical condition in which a female-bodied individual identifies as masculine. Individuals with this medical condition are called “trans men.” Complete gender transition in transmen individuals consists mainly of the following four surgical procedures: (1) chest contouring (mastectomy); (2) hysterectomy and salpingo-oophorectomy (that may be combined with mastectomy); (3) reconstruction of genital region (including vaginectomy, scrotoplasty, phalloplasty, and urethroplasty); and (4) implantation of penile prosthesis (usually 9-12 months after phalloplasty) ^7^. Therefore, a subcutaneous mastectomy (SCM) for FtM transsexuals, occasionally combined with a hysterectomy-ovariectomy, is typically the first step in the gender reassignment process after primary psychiatric evaluation and endocrine treatment 8, 9, 10. The main goal of the surgery is to masculinize the chest contour and to create an aesthetically pleasing male chest configuration.

SCM procedure includes the surgical removal of the whole gland tissue and the surplus of subcutaneous fat, tissue, and skin. Apart from the volume of the breast, the size and proper position of the nipple-areola complex (NAC) are important factors to consider in achieving a satisfactory aesthetic result^11^, ^12^, ^13^, ^14^. Four major procedures for SCM in FtM transsexuals have been described in the literature: through a semi-circular, a concentric circumareolar incision, inferior pedicled mammaplasty, with inframammary skin resection, and inframammary skin resection with full-thickness free nipple graft^8^^,^^15^. The most important factors for choosing the optimal surgical technique are the grade of skin excess or ptosis, breast size, and skin quality or elasticity^9^^,^^16^.

Some authors report complication rates after SCM in FtM individuals of up to 33%, with postoperative bleeding being the primary complication and hematoma being the most common reason for acute reoperation^16^, ^17^, ^18^. However, data analysis of postoperative bleeding and reoperation rates between different surgical techniques is lacking. Therefore, it remains unclear in which cases and which surgical techniques for SCM in FtM individuals should be prioritized, especially when a preoperative condition leading to a higher bleeding risk is known.

Our study aimed to analyze the relationship between surgical techniques, risk factors, and postoperative complications, including acute reoperations. Furthermore, we aimed to develop an algorithm specifically designed to assist clinicians in making more informed decisions regarding the surgical technique, particularly in cases where a known preoperative condition leads to a higher bleeding risk.

## Materials and Methods

In this retrospective cross-sectional study, we investigated FtM transsexuals who underwent bilateral SCM between June 2014 and June 2022. All patients have undergone masculinizing hormone therapy for at least one year, and psychiatric evaluation confirmed transsexual identity. The clinical and demographic data needed for the study were collected from digital database of patient records.

### Surgical techniques

Four different surgical techniques were used to perform subcutaneous mastectomy (Figure 2). The appropriate technique was selected according to the breast size, grade of mastoptosis, amount of skin envelope and its elasticity, and NAC diameter. In cases of small breasts (A Cup), mastoptosis grade 0-1 by Regnault^19^ and good skin elasticity, an SCM was performed through a semi-circular incision (SCI). In cases with medium breasts (B Cup), mastoptosis grade 0-1 by Regnault was performed by an SCM through a concentric circumareolar incision (CCI). In this case, a purse-string suture was performed circumferentially, and areolar diameter was reduced to 2.5-3 cm. In cases with large breasts (C Cup) and mastoptosis grade 2-3 with moderate skin elasticity, an inferior pedicled mammaplasty with inframammary skin resection (IPM) was performed (Figure 3). In cases with large breasts with poor skin elasticity and very large breasts (D Cup or bigger), an inframammary skin resection and a full-thickness free nipple graft (FTNG) (2.5-3 cm in diameter) were performed (Figure 4). Histopathological examination of all resected samples was routinely performed. Microsurgical loupes with headlamps were used as standard in all surgeries. Additionally, a lighted breast retractor was employed in cases of small incisions. All intraoperatively placed drains were removed when drainage decreased below 30 ml within 24 h and showed no abnormalities in fluid consistency or color. All patients were mobilized on the first postoperative day by physiotherapists and directly received postoperative anticoagulation prophylaxis with low-dose heparin. Postoperatively, all patients wore a compression garment for at least 6 weeks to allow optimal wound healing and reduce the risk of seroma and hematoma formation.

### Statistical analysis

Values are shown as mean ± standard deviation. Categorical variables are reported as numbers and percentages. Statistical analysis was performed using SPSS version 29.0 (IBM® SPSS®-Softwareplattform for Windows). A value of p ≤ 0.05 was considered significant. Spearman's correlation was used to find the correlation between continuous variables. Dichotomous variables were compared using Pearson's chi-squared test and the z-test for independent proportions.

## Results

We included 22 patients, with 44 subcutaneous mastectomies performed between June 2014 and September 2023. Inferior pedicled mammaplasty with inframammary skin resection and free nipple graft was performed most frequently (n = 12, 54.5%), followed by SCM through a CCI (n = 5, 22.7%) (Table 1). The chosen technique correlated strongly with removed breast weight (r = 0.66, p < 0.001). Removed breast weight was significantly associated with higher BMI (r = 0.72, p < 0.001) as well as grade of mastoptosis (r = 0.55, p = 0.008), but not age at the time of surgery (p = 0.43), duration of surgery (p = 0.25), or duration of hospital stay (p = 0.38).

**Table 1:** Relation between surgical techniques and age, weight of resected breast tissue, duration of operation, and hospital stay.

Surgical technique (number of breasts operated)	Semi-circular incision (n = 6)	Concentric circumareolar incision (n = 10)	Inferior pedicled mammaplasty with inframammary skin resection (n = 4)	Inframammary skin resection with full-thickness free nipple graft (n = 24)	Total (n = 44)
Age at the time of surgery (years)	26.3 ± 4.0	27.2 ± 7.8	17.5 ± 2.1	22.4 ± 7.5	23.6 ± 7.2
BMI (kg/m²)	24.1 ± 2.1	23.7 ± 1.7	22.6 ± 0.8	27.8 ± 6.3	25.9 ± 5.1
Weight of resected breast tissue (g)⁎	200.7 ± 135.5	201.2 ± 191.6	416.5 ± 84.2	1167.9 ± 662.0	748.0 ± 681.1
Duration of hospital stay (days)⁎⁎	4.3 ± 1.5	3.0 ± 1.0	6.5 ± 2.1	6.8 ± 3.8	5.6 ± 3.3
Duration of operation (min)	129.3 ± 9.7	168.6 ± 63.4	175.0 ± 67.9	167.8 ± 48.6	163.4 ± 49.2

⁎ Statistically significant difference in weight of resected breast tissue between IPM and other techniques except for FTNG (vs. SCI, p = 0.03; vs. CCI, p = 0.01);

⁎⁎ Statistically significant difference in the duration of hospital stay between SCM through CCI vs. IPM (p = 0.02).

The average duration of surgery was 163.4 ± 49.2 minutes. There were no statistically significant differences in the duration of surgery between surgical techniques (Table 2). There was no significant difference in the duration of hospital stay between surgical techniques, except for CCI vs. IPM (3.0 ± 1.0 vs. 6.5 ± 2.1 days, p = 0.02) (Table 3).

**Table 2:** P-values for the comparison of surgical techniques in terms of the duration of surgery.

	SCI	CCI	IPM	FTNG
SCI	-	0.34	0.30	0.21
CCI	0.34	-	0.91	0.98
IPM	0.30	0.91	-	0.85
FTNG	0.21	0.98	0.85	-

Semi-circular incision (SCI), concentric circumareolar incision (CCI), inferior pedicled mammaplasty with inframammary skin resection (IPM), inframammary skin resection with full-thickness free nipple graft (FTNG).

**Table 3:** P-values for the comparison of surgical techniques in terms of the duration of hospital stay.

	SCI	CCI	IPM	FTNG
SCI	-	0.74	0.27	0.05
CCI	0.74	-	0.02	0.05
IPM	0.02	0.02	-	0.93
FTNG	0.05	0.05	0.93	-

Semi-circular incision (SCI), concentric circumareolar incision (CCI), inferior pedicled mammaplasty with inframammary skin resection (IPM), inframammary skin resection with full-thickness free nipple graft (FTNG).

The association between surgical technique and the total number of acute reoperations caused by acute complications is demonstrated in Table 2. Acute complications were grouped into seroma and wound dehiscence (n = 1, 2.3%) and postoperative bleeding (n = 9, 20.5%). Every patient needed revision surgery because of bleeding. Patients who experienced an acute complication requiring subsequent reoperation had longer hospital stays, although the difference was not statistically significant (7.1 ± 4.5 vs. 4.5 ± 1.6 days, p = 0.06). BMI, breast weight, and duration of surgery were not associated with the rate of acute complications (p = 0.45, p = 0.57, and p = 0.08, respectively). The number of acute postoperative bleeding was highest in patients following an SCI (n = 2, 33.3%) (Table 4). The acute complication rate did not differ between patients who did and did not undergo inferior pedicled mammaplasty with inframammary skin resection (18.8% vs. 21.4% overall, p = 0.81). There was also no significant difference in frequencies of acute postoperative bleeding between surgical techniques (Table 5). There were no nipple graft necroses. One patient who received a CCI mastectomy experienced seroma and wound dehiscence, which was successfully treated under conservative therapy. One of the patients underwent a combined surgery with hysterectomy and salpingo-oophorectomy and was not associated with any postoperative complications.

**Table 4:** Rates of acute postoperative bleedings per breast between surgical techniques.

Surgical technique (number of breasts operated)	Semi-circular incision (n = 6)	Concentric circumareolar incision (n = 10)	Inferior pedicled mammaplasty with inframammary skin resection (n = 4)	Inframammary skin resection with full-thickness free nipple graft (n = 24)	Total (n = 44)
Number of acute postoperative bleedings per breast⁎	2 (33.3%)	1 (10.0%)	1 (25%)	5 (20.8%)	9 (20.5%)

⁎ There were no statistically significant differences in frequencies of acute postoperative bleeding between the techniques.

**Table 5:** P-values for the comparison of surgical techniques in terms of rates of acute postoperative bleedings.

	SCI	CCI	IPM	FTNG
SCI	-	0.18	0.71	0.83
CCI	0.18	-	0.44	0.38
IPM	0.71	0.44	-	0.83
FTNG	0.83	0.38	0.83	-

Semi-circular incision (SCI), concentric circumareolar incision (CCI), inferior pedicled mammaplasty with inframammary skin resection (IPM), inframammary skin resection with full-thickness free nipple graft (FTNG).

Patients were asked at discharge about their satisfaction with the postoperative results. The survey revealed that all patients were satisfied with the results and would undergo the same surgery again.

## Discussion

To optimize the aesthetic results, minimize complication rates, and increase the surgery's success rates and satisfactory outcomes, the surgical technique should be chosen individually in every case. The most important factors for selecting the optimal subcutaneous mastectomy surgical technique remain the grade of skin excess or ptosis, breast size, and skin quality or elasticity^8^, ^9^, ^10^.

In larger studies of patients with FtM, overall complication rates range between 10% and 35%^13^. Reoperation rates because of acute complications vary in the literature between 4% and 30%^8^^,^^9^^,^^15^^,^^16^^,^^20^, ^21^, ^22^. We reported overall complication rates of 22.7%, a little higher than reported in the literature. Most of these were postoperative bleeding with hematoma formation—20.8% of all operated breasts. The highest number of acute complications was registered in patients undergoing SCI mastectomy. Many authors also report more acute postoperative bleedings with hematoma formation in FtM individuals who underwent small incisions for SCM, namely the CCI and SCI groups^13^^,^^16^^,^^20^. Although the rates of acute revisions in these patients differ between centers, some authors prefer a conservative treatment and mention acute revision rates as low as 50% of all postoperative bleedings^20^. On the other hand, other authors, as well as we in our center, prefer revision surgery in all patients with acute postoperative bleeding with hematoma formation^8^.

Postoperative bleeding rates after SCM in transgender patients are significantly higher compared with oncological mastectomy, ranging from 3% to 5%^23^^,^^24^. This could be attributed to the limited visibility of the surgical field. Breast masculinization, seen from a surgical perspective, is primarily an aesthetic procedure necessitating smaller incisions. In this context, smaller incisions are more important than oncological surgery, where scar-sparing approaches might impede thorough tumor tissue removal. Using small incision techniques may compromise achieving proper hemostasis in a highly vascular area, even with prophylactic measures like microsurgical loupes with headlamps, illuminated breast retractors, or artificially elevating blood pressure before wound closure. Similar surgical techniques are employed in SCM for patients with gynecomastia, and the literature reports also higher complication rates ranging from 3% to 28%^25^^,^^26^. Hematomas and seromas are the predominant postoperative complications in these patients ^26^.

Several authors discussing FtM individuals have suggested a potential influence of sexual hormones on hemostasis^13^. It is known that higher levels of serum testosterone beyond the physiological range for males can lead to vaginal bleeding and spotting in these patients^27^. Furthermore, there is some evidence suggesting that testosterone therapy may impact the fibrinolytic system, potentially exerting antithrombotic effects on hemostasis^28^. Therefore, preoperative hormone therapy could play a key role in the observed high hematoma rates. However, further studies are needed to confirm these effects. Patients who experienced an acute complication and required subsequent revision surgery tended to have longer hospitalizations, although we could not identify a statistical significance. A prolongation of hospital stay following acute complications has been previously mentioned in other studies^8^. However, it seems that the length of stay after the revision surgery mostly depends on the clinic's general policy.

We constantly improve the perioperative care for this special patient population, and as a breast center, we incorporate proven optimizing measures to reduce complication rates. For example, we artificially elevate blood pressure before wound closure to a systolic pressure of 130 mmHg. Additionally, we prescribe a distinct postoperative regimen (including bedrest, the consequent wearing of compression bandage postoperatively for at least 24 h, and analgetic therapy with Metamizole instead of Ibuprofen)^29^^,^^30^. We have recently started using tranexamic acid in patients with a tendency for bleeding, such as frequent nasal bleedings or a history of bruising easily, following the discovery by Devereaux et al. that in patients undergoing noncardiac surgery, the incidence of the composite bleeding outcome could be significantly reduced^31^.

The patients who received an SCM with full-thickness free nipple grafting are at risk for total or partial nipple graft necrosis. The published data shows the risk for that complication is as high as 2.3% to 17.9%^20^. In our study group, we had no FTNG revisions. Our technique of free nipple grafting is combined with a strict postoperative regimen, including bolster dressings always supported by compression garments, and keeping patients in bed until the first mobilization with the support of physiotherapists has proven to be very effective in preventing complications of free nipple graft. Moreover, we perform FtM chest wall procedures always as an inpatient operation to better control the early postoperative phase. We believe this strict regimen is responsible for these favorable results of free nipple grafting.

One patient who received a CCI mastectomy experienced seroma and wound dehiscence on one side without the need for surgical treatment. According to recent data, complications such as seroma and wound dehiscence are more common in smaller incision techniques such as semi-circular or concentric circumareolar. Published rates lie between 13% and 18%^21^^,^^32^. All the patients in our clinic remain hospitalized until all the drains are removed on the third or even fourth postoperative day. Therefore, wound management, as well as change of wound dressings on the first days after surgery, are done by specialized personnel, which may lead to fewer wound healing-associated complications. In our center, we prefer a longer hospital stay after an SCM, especially after a SCI, to monitor the wound healing closely, early detect any possible deviations from normal postoperative course, and therefore reduce acute complications rates.

Satisfaction with the postoperative results is an important factor in FtM individuals. It is proven that SCM in these patients has a positive psychological effect that leads to a higher quality of life and self-esteem^33^. All our patients expressed high satisfaction with the postoperative results, although we have not performed any standardized questionnaire. Other authors report also very high patients’ satisfaction^16^^,^^34^. On the other hand, a review published by Cohen et al. states that there is no unified system for evaluating patient satisfaction with the results of surgery after chest contouring^13^. Therefore, a comparison and interpretation of these evaluations is still limited.

Despite the valuable insights gained from our study, several limitations must be acknowledged in the present investigation. Foremost among these limitations is the relatively low number of patients included in the study cohort. The limited number of patients hampers the statistical power of the study, which, in turn, potentially restricts the ability to draw definitive conclusions and discuss significance with highest confidence. Furthermore, because of the retrospective study design, causal relationships cannot be established definitively, even though significant tendencies were identified from the observed data. Despite the limitations, this study offers valuable preliminary findings of the higher risk of bleeding complications after SCM in FtM transgender population, which will serve as a foundation for future investigations.

## Conclusions

In summary, our study revealed that less invasive techniques are associated with higher rates of postoperative bleeding. We could not identify any perioperative risk factors significantly associated with increased complication rates. Many authors in literature have discussed the potential impact of preoperative hormone therapy on compromising hemostasis in these patients, potentially increasing the risk of bleeding. However, the existing data on the relationship between hormone therapy and coagulation are inconclusive, leaving this question unanswered. Therefore, the primary focus continues to be on surgical techniques and perioperative management.

**Figure 1:**
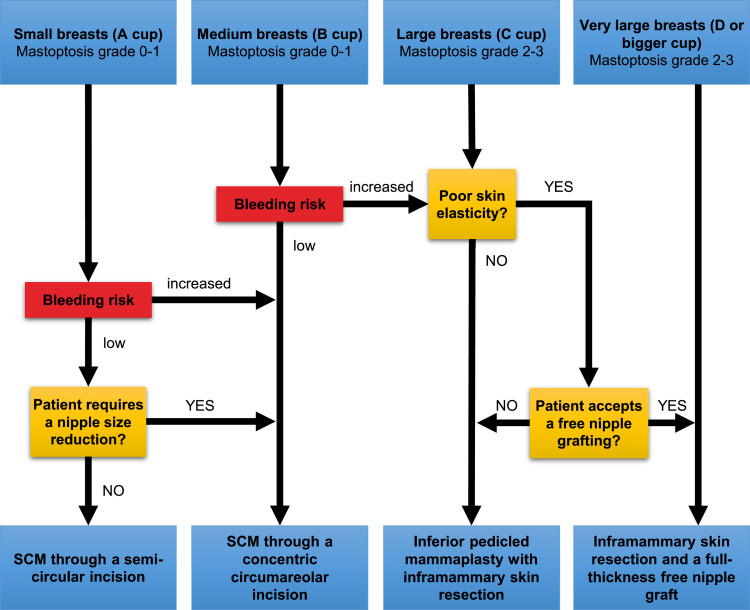
Algorithm for surgical techniques of SCM in FtM transsexuals regarding the individual risk of bleeding.

Gender reassignment surgery, particularly subcutaneous mastectomy, presents unique challenges for the surgeon, given its aesthetic nature. Patient preferences for smaller incisions to minimize postoperative scarring can result in reduced visibility of the surgical field and additional challenges in achieving proper hemostasis. Based on our data and experience, we recommend prioritizing techniques with improved visibility of the surgical field in FtM individuals with comorbidities that may increase the bleeding risk. We have developed an algorithm to assist clinicians in making informed decisions regarding surgical techniques in such cases (Figure 1).

**Figure 2:**
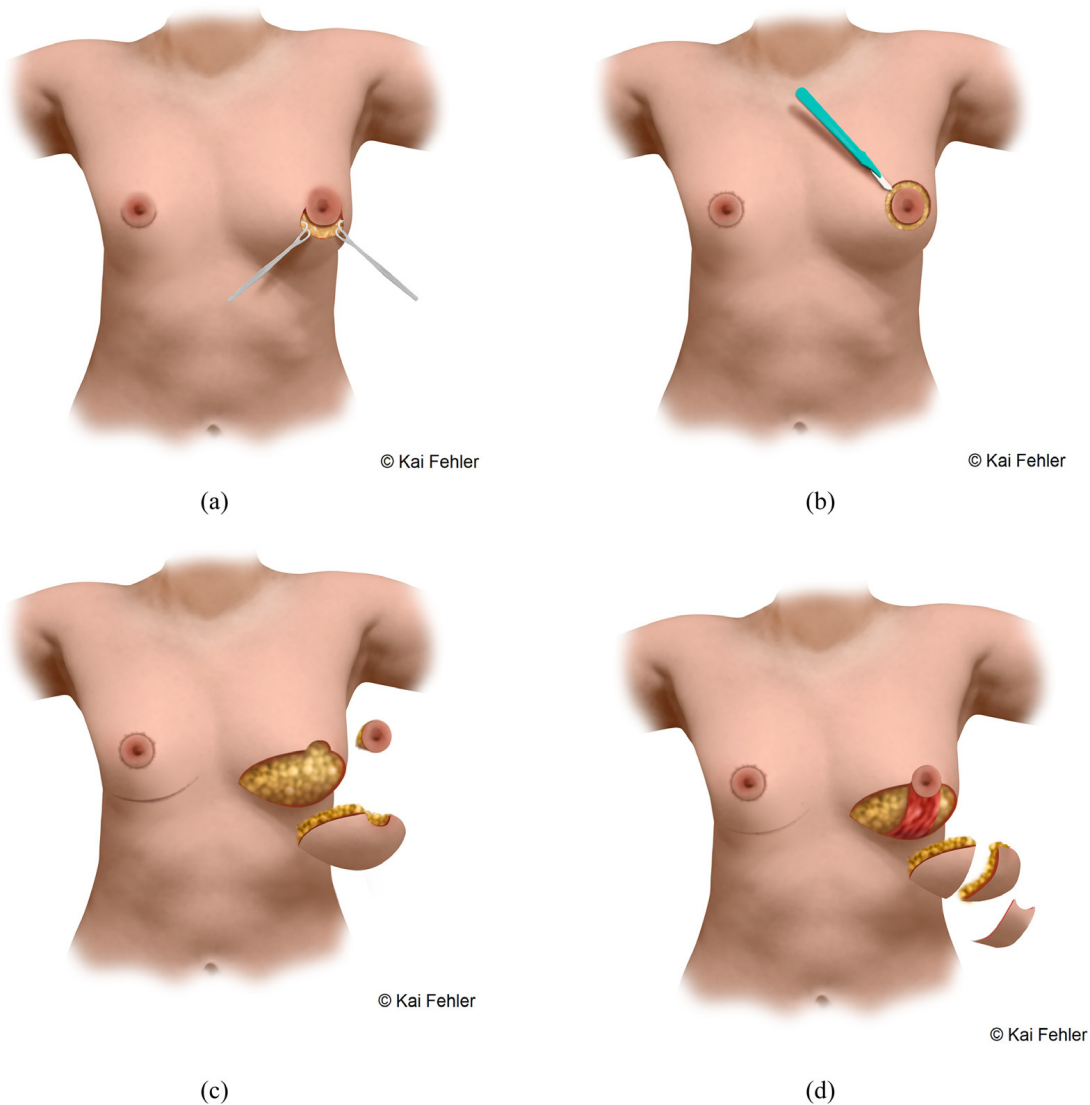
Surgical techniques of subcutaneous mastectomy (Illustrations created by Kai Fehler). A: SCM through a semi-circular incision (left breast: operation technique; right breast: postoperative aesthetic result). B: SCM through a concentric circumareolar incision (left breast: operation technique; right breast: postoperative aesthetic result). C: SCM through an inferior pedicled mammaplasty with inframammary skin resection and pedicled nipple (left breast: operation technique; right breast: postoperative aesthetic result). D: SCM through an inframammary skin resection and a full-thickness free nipple graft (left breast: operation technique; right breast: postoperative aesthetic result)

**Figure 3:**
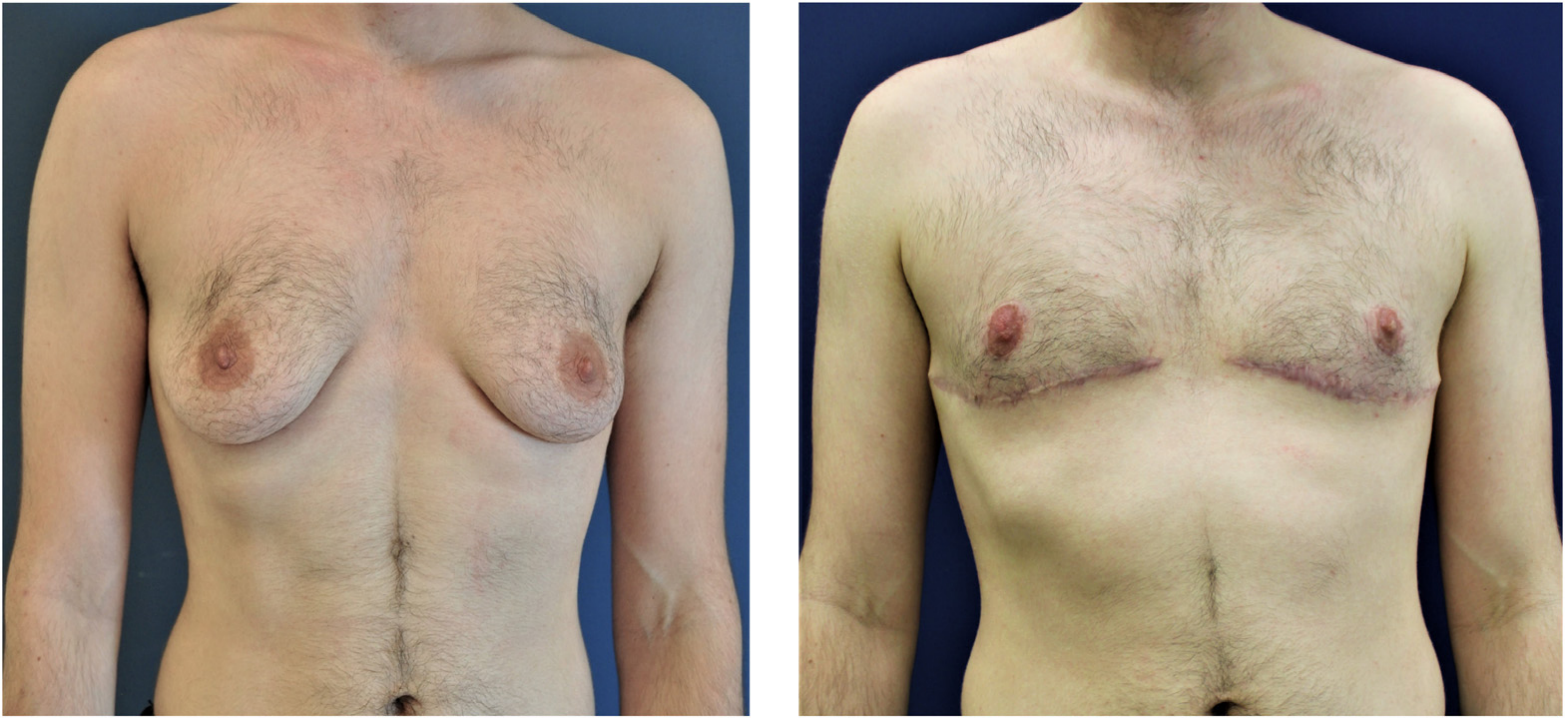
SCM through an inferior pedicled mammaplasty with inframammary skin resection and pedicled nipple before (left) and after (right) the surgery.

**Figure 4:**
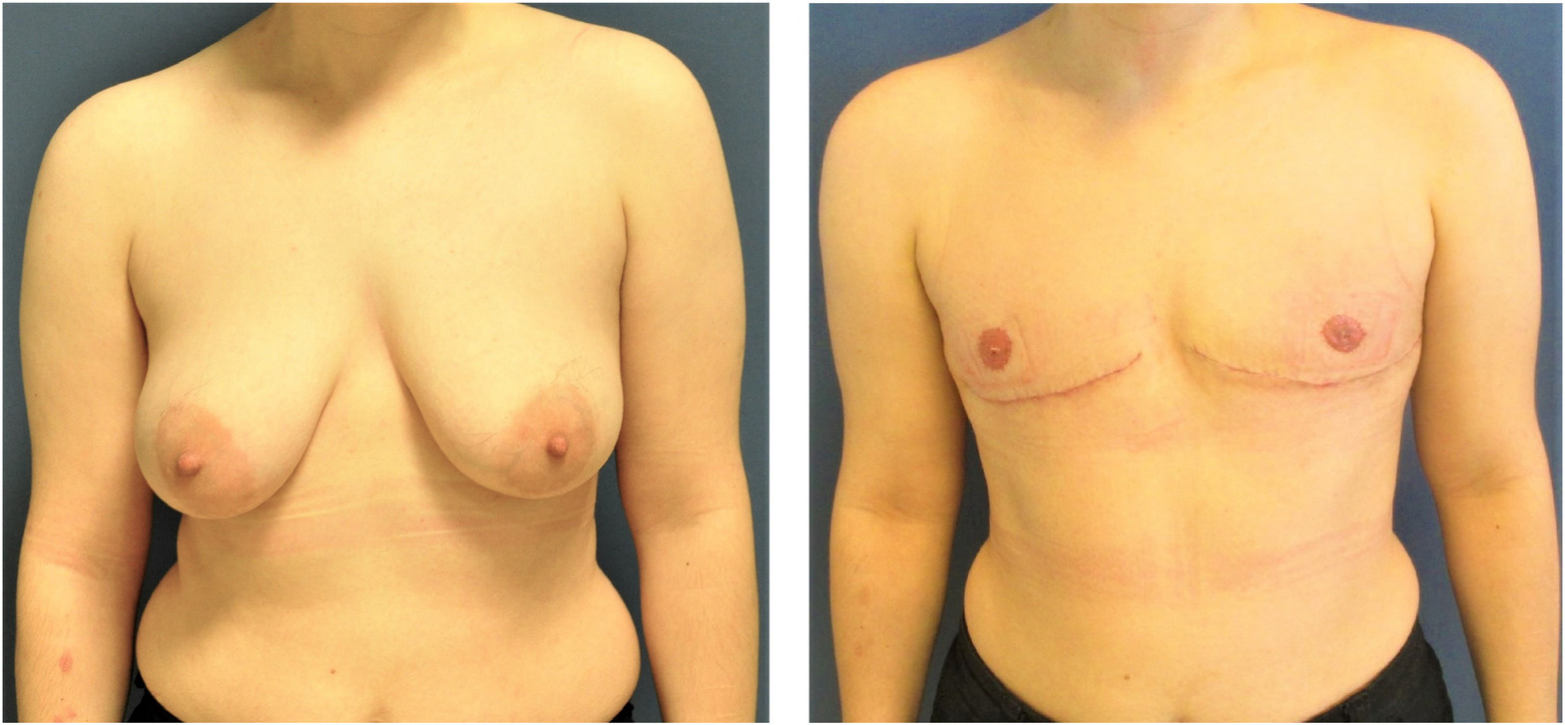
SCM through an inframammary skin resection and a full-thickness free nipple graft before (left) and after (right) the surgery.

Despite the growing experience and the availability of valuable data, gender affirmation surgery remains a developing field. Consequently, further studies investigating the influence of hormone therapy on postoperative bleeding and potential prophylactic measures are urgently needed. These studies will equip surgeons and other healthcare professionals with the necessary knowledge to effectively reduce morbidity in this patient group.
